# Significance of nuclear morphometry in cytological aspirates of breast masses

**DOI:** 10.4103/0970-9371.66694

**Published:** 2010-01

**Authors:** Shivani Kalhan, Suparna Dubey, Sonia Sharma, Sharmila Dudani, Preeti, Monika Dixit

**Affiliations:** Department of Pathology, Army College of Medical Sciences, Delhi Cantt., Delhi, India; 1 Department of Pathology, Adesh Institute of Medical Sciences and Research, Bathinda, Punjab, India

**Keywords:** Breast carcinoma, cytologic grading, fine needle aspiration cytology, nuclear morphometry

## Abstract

**Background:**

Breast carcinoma is the most common malignancy globally. Cytological evaluation in breast lesions is largely subjective. Gradual progression of cells from normal to invasive involves nuclear changes that need to be viewed objectively.

**Aims:**

This study aims to apply nuclear morphometry on cytological breast aspirates. It evaluates its utility in differentiating benign *vs*. malignant lesions and correlates it with cytologic grading in malignant cases.

**Setting and Design:**

Nuclear morphometric parameters of malignant and benign cases were compared. Parameters of malignant cases were correlated with cytologic grading.

**Materials and Methods:**

Cytology was used to categorize aspirates from breast lumps into malignant (53 cases) and benign (29 cases). One hundred cells per case in both groups were mapped on DEBEL Cytoscan and six geometrical and three textural parameters obtained were compared. In malignant cases, morphometry was correlated with Robinson’s cytologic grading, which was further correlated in tissue sections (45 cases) with modified Scarff-Bloom-Richardson histologic grading.

**Statistical Analysis:**

Students “*t*”-test was applied for comparison between benign and malignant cases. One-way ANOVA followed by Bonferroni’s *post hoc* comparison was applied to compare the three cytologic grades. Results were considered significant when *P*<0.05.

**Results:**

Nuclear morphometry successfully differentiated between benign and malignant aspirates and correlated significantly with cytologic grades. Morphometry was especially useful in the diagnosis of atypical ductal hyperplasia and ductal carcinoma *in situ*. Useful parameters were mean nuclear area, long axis, short axis and total run length. Cytohistologic correlation was 83.3%, 88.9% and 88.9% for cytological grades 1, 2 and 3 respectively.

**Conclusions:**

Nuclear morphometry was thus a useful objective tool in the evaluation of breast masses.

## Introduction

Breast carcinoma is the most common malignancy in the female population, especially in Western countries, and is now replacing cervical cancer as the leading cancer site.[1] It is also emerging as the leading cause of cancer mortality in Indian women.[2]

Fine needle aspiration cytology (FNAC) is applied as the primary tool for diagnosis in breast masses because of its ease and rapidity, but is, till date, largely subjective. The morphological overlap among the sequential lesions from the precancerous group to frank carcinoma further causes a “gray zone” in cytology, estimated to constitute 8.9%[3,4] Application of ancillary techniques like morphometry, immunohistochemistry and flow cytometry are useful in providing an objective and reproducible diagnosis, especially in borderline and malignant lesions.[5]

Morphometry is the measurement of various cell parameters microscopically/by flow cytometry/image analysis. Its potential in cytology is yet to be fully realised. This study strives to apply nuclear morphometry to aspirates from breast masses such that the entire spectrum of pathological lesions may be studied. The parameters studied were evaluated to delineate benign from malignant lesions. The parameters of malignant cases were further compared with cytologic nuclear grading. Cytohistologic correlation was then performed.

## Materials and Methods

This was a prospective study conducted on patients presenting with breast masses in our institution over a 1-year period. A concise clinical history, examination and details of relevant investigations were also obtained. Fifty-three malignant and 29 benign cases were studied. Histopathology material was available in 45 malignant cases, which included three cases of ductal carcinoma *in situ* (DCIS). Histopathology was available in all 29 benign lesions, which included four cases of atypical ductal hyperplasia (ADH). Infiltrating duct carcinoma (IDC) cases were graded on histology as per Nottingham’s modification of Scarff Bloom Richardson’s system.

FNAC was carried out using the standard procedure. Both air-dried and alcohol-fixed smears were prepared and stained by leishman–giemsa and papanicolaou (PAP) stains, respectively. Papanicolaou-stained malignant smears were evaluated using Robinson’s cytologic grading system considering six parameters, namely cell dissociation, cell size, cell uniformity, nucleoli, nuclear margin and chromatin.[6]

Scores of 1–3 were assigned for each of the six parameters – cell dissociation, cell size, cell uniformity, nucleoli, nuclear margin and chromatin, and they were totaled to classify the lesions into: Grade 1, score 6–11; Grade 2, score 12–14; Grade 3, score 15–18.

PAP-stained smears of all cases were subjected to nuclear morphometry on a Defence Bioengineering and Electro Medical Laboratory (DEBEL) Cytoscan indigenously developed by the Defence Research and Development Organization, New Delhi, India. One hundred cells per case were evaluated. Both geometrical and textural parameters of the nuclei were studied. Geometrical parameters included nuclear area, perimeter, nuclear shape, long axis, short axis and intensity. Textural parameters were long run emphasis (measuring coarseness of nuclear chromatin), total run length (measuring proportion of coarse to fine chromatin) and T_1_ homogeneity (measuring homogeneity of chromatin distribution).

Nuclear morphometry was compared with cytologic grading in malignant cases and this was further correlated with histologic grading where tissue sections were available.

Statistical analysis was performed using SPSS version 15.0, LEAD Technologies, Inc., Charlotte, North Carolina, USA. Students “*t*”-test was applied for comparison between the benign and malignant groups. One-way ANOVA followed by Bonferroni’s *post hoc* comparison was applied to compare the three cytologic grades. Kruskal Wallis test was applied on the four groups – benign breast disease, ADH, DCIS and infiltrating carcinoma, to study the pattern of progression. Results were considered significant when *P*<0.05 and highly significant when *P*< 0.0001.

## Results

**Figure 1:**
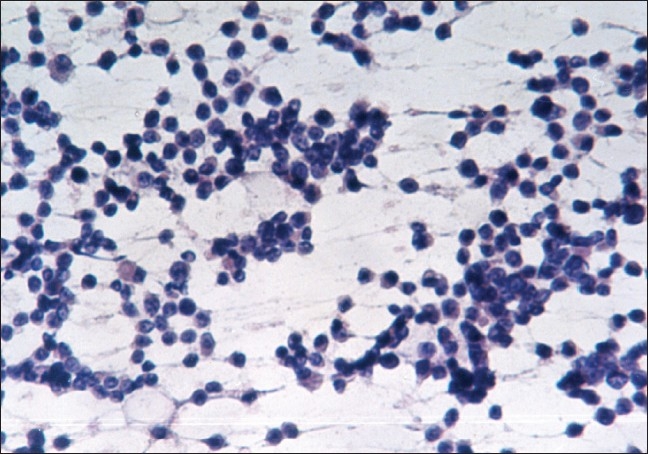
Cytological grade 1: Mixture of single cells and cell clusters (PAP, ×200)

**Figure 2:**
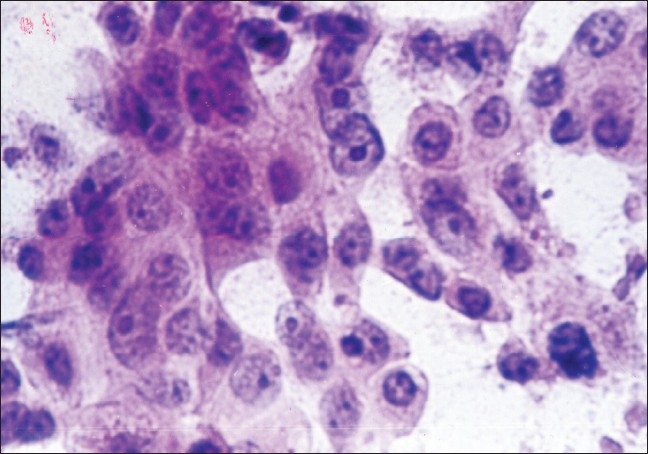
Cytological grade 2: Cells with granular chromatin and prominent nucleoli (PAP, ×400)

**Figure 3:**
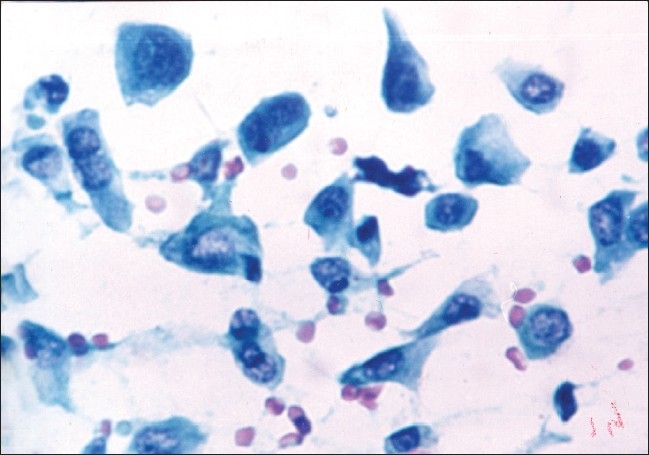
Cytological grade 3: Cell size >5 RBC and cellular pleomorphism with clefts in nuclei (PAP, ×400)

The study included 53 malignant and 29 benign cases of breast lumps as diagnosed by cytology. The patients in the malignant category included 52 females and one male. On cytologic grading of malignant cases, 11 (20.8%) were in cytological Grade 1 [Figure 1], 29 (54.7%) were clustered in the intermediate Grade 2 [Figure 2] and 13 (24.5%) in cytological Grade 3 [Figure 3].

The various geometrical and textural parameters were analysed in all cases for the critical differentiation of benign from malignant [Table 1]. Using Students “*t*”-test, the mean nuclear area, perimeter, long axis, short axis, long run emphasis, total run length and T_1_ homogeneity were found to be statistically significant (*P*< 0.05). The mean nuclear area, perimeter, long axis, short axis and total run length were highly significant, with *P*< 0.0001. Shape and intensity were found to be statistically insignificant.

**Table 1:** Morphometry: Benign *vs*. malignant lesions

Morphometric parameter	Benign (*n*=29)	Malignant (*n*=53)	*P* value
	Mean ± SD (range)	Mean ± SD (range)	
Nuclear area (sq. microns) MNA	28.46 ± 7.72 (16.9–40.59)	94.19 ± 19.49 (57.36–137.98)	<0.0001
Perimeter (microns) P	19.77 ± 2.42 (16.09–23.68)	36.19 ± 4.91 (22.41–44.88)	<0.0001
Shape (abs) S	1.08 ± 0.04 (1.036–1.15)	1.09 ± 0.04 (1.03–1.16)	0.616
Long axis (microns) LA	6.87 ± 0.87 (5.72–8.12)	13.52 ± 1.56 (10.04–15.96)	<0.0001
Short axis (microns) SA	4.95 ± 0.84 (3.91–6.32)	9.25 ± 1.24 (7.06–10.89)	<0.0001
Intensity (abs) l	114.23 ± 11.63 (100.08–131.8)	117.73 ± 19.52 (81.52–144.58)	0.314
Long run emphasis LRE	1.31 ± 0.19 (1.09–1.61)	1.20 ± 0.08 (1.09–1.35)	0.005
Total run length TRL	1172.41 ± 468.29 (644–1943.33)	4168.44 ± 1109.63 (2449.18–6341)	<0.0001
T_1_ homogeneity T_1_H	0.0067 ± 0.0003 (0.0061–0.0071)	0.0072 ± 0.0013 (0.0053–0.010)	0.005

We had four cases of ADH, which could be distinctly classified into the benign category on morphometry. We also had three cases of DCIS in which the significant morphometric parameters remained well within the malignant range, even though this is a strictly histological diagnosis.

After histology correlation, the clinically palpable masses were also categorized on cytology as benign breast disease (*n*=25), ADH (*n*=4), DCIS (*n*=3) and infiltrating carcinoma (*n*=50). The nuclear morphometric parameters showed a distinct pattern of progression in the four groups [Table 2]. Kruskal Wallis test was applied on these four groups and the parameters found significant in differentiating them were mean nuclear area (*P*< 0.0001), perimeter (*P*< 0.0001), shape (*P*=0.003), long axis (*P*< 0.0001), short axis (P0.0001), intensity (*P*=0.021), long run emphasis (*P*=0.004) and total run length (*P*< 0.0001). T_1_ homogeneity was found to be statistically insignificant (*P*=0.234).

**Table 2:** Morphometry: Progression pattern

Morphometric parameter	Benign (*n*=25)	ADH (*n*=4)	DCIS (*n*=3)	Invasive carcinoma (*n*=50)
Nuclear area	28.14 ± 7.91	30.45 ± 7.04	85.63 ± 5.10	94.71 ± 19.94
Perimeter	19.63 ± 2.48	20.62 ± 2.15	32.37 ± 2.15	36.42 ± 4.95
Shape	1.09 ± 0.04	1.04 ± 0.002	1.04 ± 0.006	1.09 ± 0.04
Long axis	6.83 ± 0.91	7.14 ± 0.53	12.02 ± 0.59	13.61 ± 1.55
Short axis	4.88 ± 0.85	5.38 ± 0.75	8.63 ± 0.54	9.28 ± 1.27
Intensity	116.32 ± 11.18	101.22 ± 0.81	92.64 ± 6.21	119.23 ± 19.03
Long run emphasis	1.34 ± 0.19	1.12 ± 0.01	1.17 ± 0.05	1.2 ± 0.08
Total run length	1116.09 ± 454.83	1524.38 ± 447.42	4043.24 ± 209.25	4175.95 ± 1141.87
T1 homogeneity	0.0066 ± 0.0003	0.0067 ± 0.0004	0.006 ± 0.0006	0.0073 ± 0.0013

ADH, atypical ductal hyperplasia; DCIS, ductal carcinoma *in situ*

To compare morphometry with cytologic grading, one-way ANOVA followed by *post hoc* comparison of multiple variables by Bonferroni’s method was performed [Table 3]. All the parameters studied, except long run emphasis, had a significant correlation with cytologic grading. Mean nuclear area, long axis, short axis and total run length showed a significant difference between the three grades.

**Table 3:** Morphometry: Cytologic grades

Morphometric parameter	Grade 1 (*n*=11)	Grade 2 (*n*=29)	Grade 3 (*n*=13)	ANOVA		*Post hoc* ANOVA *P* values		
				F-value	*P* value	1 *vs*. 2	1 *vs*. 3	2 *vs*. 3
Nuclear area	75.46 ± 12.92	92.38 ± 13.73	114.09 ± 17.59	21.34	<0.0001	0.006	<0.0001	<0.0001
Perimeter	31.27 ± 3.91	36.67 ± 3.84	39.28 ± 4.94	11.55	<0.0001	0.002	<0.0001	0.194
Shape	1.05 ± 0.01	1.10 ± 0.04	1.10 ± 0.03	10.99	<0.0001	<0.0001	<0.0001	1.000
Long axis	11.68 ± 1.07	13.66 ± 1.24	14.75 ± 1.06	21.13	<0.0001	<0.0001	<0.0001	0.022
Short axis	8.10 ± 0.81	9.23 ± 1.15	10.27 ± 0.85	13.42	<0.0001	0.009	<0.0001	0.011
Intensity	101.87 ± 18.13	119.14 ± 16.61	127.10 ± 19.61	6.71	0.003	0.024	0.002	0.419
Long run emphasis	1.21 ± 0.08	1.20 ± 0.08	1.20 ± 0.05	0.12	0.89	1.000	1.000	1.000
Total run length	3292.95 ± 787.83	4135.18 ± 1029.54	4983.42 ± 959.31	9.11	<0.0001	0.053	<0.0001	0.035
T1 homogeneity	0.0061 ± 0.0004	0.0079 ± 0.0013	0.0065 ± 0.0003	16.65	<0.0001	<0.0001	1.000	<0.0001

There were three cases of DCIS, which were classified as malignant on cytology in cytological Grade 1 and corroborated by morphometry. These were not considered while performing cytohistopathological correlation.

Cytologic grading showed a high correlation with histologic grading in the 42 cases of IDC in which tissue diagnosis was available. The overall agreement was 88.1% [Table 4]. Grade 1 showed a correlation of 83.3%, while both Grades 2 and 3 showed a concordance of 88.9%.

**Table 4:** Cytohistopathological correlation

Cytologic grade	Histopathologic grade			Total
	I	II	III	
1†	5	1	0	6*
2†	1	24	1	26
3†	0	2	8	10
Total	6	27	9	42

* Three cases of DCIS classified in histopathological Grade 1 not considered in cytohistopathological correlation.

† Tissue not available in two cases (Grade 1), three cases (Grade 2) and three cases (Grade 3)

Correlation of the three cytologic grades was performed with mitotic activity (as seen on cytology smears), tumor size and lymph node status (on histopathology material). Tumor size showed an increase with increasing cytologic grades, i.e. tumour size <5 cm was found in nine (100%) cases in Grade 1, 22 (84.6%) in Grade 2 and seven (70%) in Grade 3. Six (66.7%) of Grade 1 cases were node-negative, while a majority of 18 (69.2%) Grade 2 cases showed positivity in one to three nodes and a majority of five (50%) Grade 3 cases showed positivity in >4 nodes. Lymph node positivity again showed an increase with increasing cytologic grades.

## Discussion

Breast lesions account for one of the largest group of conditions necessitating pathological, radiological and surgical intervention. Breast carcinoma is emerging as the most common malignancy globally. In India, the cervix cancer rates are decreasing while breast cancer is on the increase, especially in urban areas.[1] Breast carcinoma is emerging as the leading cause of cancer mortality in Indian women, with nearly 80,000 new cases of breast cancer being diagnosed annually in India.[2]

FNAC is the first diagnostic modality employed for the diagnosis of breast masses. However, cytology has its own disadvantages, like interobserver and intra-observer variability. It is further compounded by the morphological overlap among the sequential lesions from the precancerous group to frank carcinoma. The progression from normal breast to ductal hyperplasia and ADH going into DCIS and invasive carcinoma (with or without metastasis) reveals sequential events.[3] This “gray zone” in cytology is estimated to constitute 8.9% of cases. This encompasses three categories – technical limitations (4.5%), inexperience of the cytopathologist (2.4%) and overlap of cytological features of benign *vs*. malignant (2%).[4]

Nuclear grading was first introduced by Black *et al*.^7^ and refined by various workers till a composite cytonuclear grading system was introduced by Robinson *et al*.,[6] which has been used in this study.

In this era of automation, this study introduces morphometry as a highly objective tool to supplement the entirely subjective FNAC in the crucial differentiation of benign from malignant lesions. Further evaluation regarding its role in grading the malignant breast aspirates in relation to the already established cytologic grading was attempted. Special emphasis was laid on the correct classification of the borderline cases in the gray zone comprising of ADH and DCIS on morphometric evaluation.

Alterations in nuclear structure are the morphologic hallmarks of cancer diagnosis.[8] This study has thus focused on nuclear morphometry in breast tumors. Morphometry in breast tumors has been studied by a number of workers on histological sections[8–13] and on cytology.[9,14–21] A large number of parameters have been studied by morphometry, but the nuclear parameters related to nuclear size, like area, perimeter, diameter or axes, have consistently been found to be significant, both in histology and cytology, in distinguishing benign *vs*. malignant lesions. Mean nuclear area is the most consistent of them all.[5,10,16,19,21–23]

In our study, similarly, we found all parameters except shape and intensity to be significant in differentiating benign from malignant lesions. Of these, geometrical parameters: mean nuclear area, perimeter, long axis and short axis and textural parameter: total run length were highly significant (*P*< 0.0001). Moreover, the highly significant parameters, except perimeter, showed no overlap whatsoever among the benign and malignant aspirates. Of special note is the observation that the four cases of ADH and three cases of DCIS could be distinctly classified into the benign and malignant categories, respectively, by these parameters, even though these are strictly histological diagnoses. This is a relevant finding, which will help in categorizing the gray zone objectively.

Mean nuclear area is the most studied parameter in nuclear morphometry in the published literature.[5,10,16,19,21–23] Our benign cases showed a mean nuclear area <40.59 sq. microns and malignant ones had a mean nuclear area >57.36 sq. microns, which is well in agreement with the other studies[17,18,21]

The progression pattern of nuclear morphometric parameters has been emphasised in various studies,[8,9,13,21,23–25] with gradually increasing values from benign to atypical, DCIS and further to invasive carcinoma and carcinoma with lymph node involvement. All these studies have been carried out on histopathology material. Our study, based on cytology, has brought out the gradual increase in the mean numerical values of the parameters like mean nuclear area, perimeter, long, short axis and total run length.

Some studies have explored the correlation between morphometry and cytologic grading using various morphometric parameters.[9,16,17] Most have found a significant association using multivariate analysis.[24,25] However, in our study, although there is an overlap between the various grades, all the parameters studied, except long run emphasis, had a significant correlation with cytologic grading on ANOVA test. On application of *post hoc* ANOVA, however, it was found that perimeter, shape and intensity could be used to differentiate between Grades 1 *vs*. 2 and between Grades 1 *vs*. 3. These were not significant in differentiating between Grades 2 *vs*. 3. T_1_ homogeneity, on the other hand, showed a significant difference between Grades 1 *vs*. 2 and Grades 2 *vs*. 3. Mean nuclear area, long axis, short axis and total run length showed a significant difference between the three grades, and any of these could be used in univariate analysis to differentiate between the three cytological malignant grades by automation. This is a significant finding, which sets this study apart and emphasizes the immense scope of morphometry.

Tissue diagnosis was available in 45 of the 53 malignant cases. It was also available in all 29 cases of the benign category, including four cases of ADH. Cytohistological correlation was performed in 42 IDC cases. Thirty-seven cases had matching cytohistological grades, giving an overall concordance of 88.1%. Cytohistological correlation has been found to vary from 57.1% to 95% in the literature,[26,27] and our study lies at the higher end of the spectrum. A recent study showed an overall and cytological grade-wise concordance rate comparable to our study.[28]

An attempt was made to also study the clinicopathological features of the tumors in the malignant group by correlating cytologic grade with tumor size and lymph node status on the histopathology material and mitotic count per slide on the cytological material. Tumor size, node positivity and mitotic count showed an increase with increasing cytologic grades and morphometric parameters in this study. The progressive values in morphometry have been found to correlate with tumor size,[10,11,24,25] lymph node involvement[10,24] and mitotic activity[11,16] in various studies and have been used to predict prognosis.[24,25] Therefore, the impact of clinicopathological features along with morphometric parameters should be evaluated to determine the aggressiveness of the tumor.

## Conclusions

Morphometry has been found to be valuable in differentiating benign lesions from malignant ones on cytology in this study. It has been proved to be a useful objective tool, especially in the “gray zone”, where diagnostic dilemmas are encountered. Despite the limitation of small sample size, our study indicates that nuclear morphometry may be applied to delineate lesions like ADH and DCIS. However, this needs further study on a larger number of cases. This modality can also be put to use in far-flung areas or areas where technical expertise is limited.

Nuclear morphometry can further be applied to augment the cytological grading of breast cancer. The parameters found to be consistently useful are mean nuclear area, long axis, short axis and total run length. These, coupled with other clinicopathological features such as tumor size, lymph node positivity and mitotic activity, may be used to prognosticate and classify the patients into low- and high-risk groups.
